# All That Coughs Is Not COVID

**DOI:** 10.7759/cureus.32169

**Published:** 2022-12-03

**Authors:** Nisha M Patel, Matthew Shaines, Priya Nori

**Affiliations:** 1 Internal Medicine, Montefiore Medical Center/Albert Einstein College of Medicine, Bronx, USA; 2 Hospital Medicine, Montefiore Medical Center/Albert Einstein College of Medicine, Bronx, USA; 3 Infectious Diseases, Montefiore Medical Center/Albert Einstein College of Medicine, Bronx, USA

**Keywords:** historically marginalized populations, social determinants of health (sdoh), febrile respiratory illness, community acquired pneumonia, legionnaires’ disease, legionella, covid

## Abstract

We report the case of a woman from the Bronx, New York, who presented to the emergency department (ED) in June 2020 with a febrile respiratory illness resembling coronavirus disease 2019 (COVID-19) but was ultimately diagnosed with Legionnaires’ disease (LD). New York City (NYC) rapidly became an epicenter of the global COVID-19 pandemic in 2020. In the years since the pandemic started, variants of severe acute respiratory syndrome coronavirus 2 (SARS-CoV-2) have recurred in multiple waves and remain an important cause of viral respiratory illness. The bacteria *Legionella pneumophila* is often under-recognized as a cause of community-acquired pneumonia, yet it recurs each year in clusters, outbreaks, or as sporadic infections. Pneumonia caused by SARS-CoV-2 and *Legionella *can present similarly and may not be readily distinguished in the absence of diagnostic testing.

## Introduction

Legionellosis refers to the disease caused by the bacterial genus *Legionella.* It can present as an influenza-like illness (Pontiac fever) or bacterial pneumonia, referred to as Legionnaires’ disease (LD) [1]. The clinical manifestations of LD include fever, myalgias, headaches, dyspnea, cough, chest pain, and diarrhea [2]. The presenting symptoms of LD overlap with viral respiratory illnesses such as influenza and COVID-19. The latter does not appear to have an organized seasonal pattern and has occurred in waves in both summer and winter months, unlike LD, which more predictably occurs during warmer, humid months. Due to its nonspecific clinical presentation, the diagnosis of LD requires microbiologic testing that may not be performed routinely [1], therefore, clinical suspicion for LD must remain high and a detailed social history, including epidemiologic risk factors, must be obtained.

While the COVID-19 pandemic is relatively new, it has had a clear, disproportionate impact on people living in poverty and those from historically marginalized racial and ethnic groups [3]. Although LD is not a novel disease, it continues to occur yearly since its emergence in 1976 and has the highest impact on these same communities [4].

## Case presentation

A 58-year-old woman presented with three days of fevers and shortness of breath to an emergency department (ED) in the Bronx, NY, in June 2020. She had a history of human immunodeficiency virus (HIV; with intermittent adherence to antiretroviral therapy), asthma, and a 40-pack-year history of cigarette smoking. She reported accompanying anorexia, generalized weakness, and a slight dry cough. She denied chest pain, abdominal pain, nausea, vomiting, or diarrhea. She used her asthma medications regularly at home with only temporary relief of dyspnea, which prompted her visit to the hospital.

On presentation, her temperature was 102.9°F, blood pressure 174/94 mmHg, heart rate 119 beats per minute, respiratory rate 20 breaths per minute, and oxygen saturation 98% on ambient air. Her physical exam was notable for expiratory wheezes in the left middle and lower lung fields. There was no elevated jugular venous pressure or lower extremity edema.

**Figure 1:**
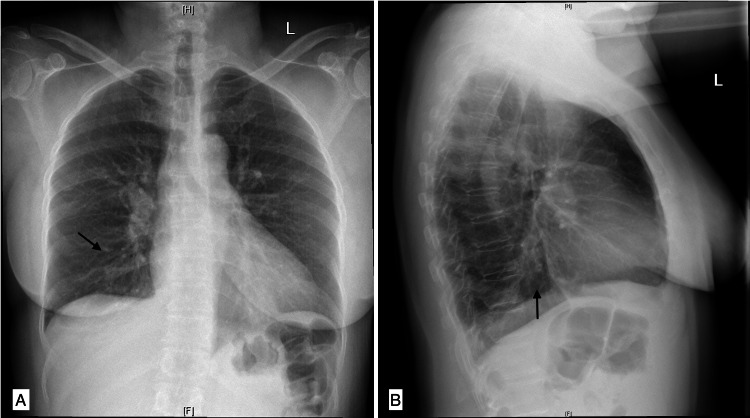
Initial chest radiography A) posterior-anterior view; B) lateral view Arrows indicate subtle lower lobe opacities.

A chest X-ray showed subtle bilateral lower lobe opacities, as depicted in Figure 1 below.

SARS-CoV-2 polymerase chain reaction (PCR) of a nasopharyngeal specimen was negative. Other notable laboratory tests are depicted in Table 1.

**Table 1:** Patient’s laboratory values on initial presentation HIV: human immunodeficiency virus; RNA: ribonucleic acid; CD4: cluster of differentiation 4

Laboratory Test	Results	Normal Range	Interpretation
White blood cell count	12.4 k/uL	4.8 - 10.8 k/uL	Increased
Neutrophil count	10.4 k/uL	1.8 – 7.7 k/uL	Increased
Lymphocyte count	1.4 k/uL	1.8 – 4.8 k/uL	Normal
Monocyte count	0.5 k/uL	0.3 – 0.5 k/uL	Normal
Eosinophil count	0.0 k/uL	0.1 – 0.3 k/uL	Decreased
Basophil count	0.02 k/uL	0.0 – 0.05 k/uL	Normal
Serum sodium	128 mEq/L	135 - 145 mEq/L	Decreased
Blood urea nitrogen	7 mg/dL	5-20 mg/dL	Normal
Creatinine	0.8 mg/dL	<1.20 mg/dL	Normal
D-dimer	2.13 ug/mL FEU	0.00 – 0.50 ug/mL FEU	Increased
C-reactive protein	32.6 mg/dL	<0.8 mg/dL	Increased
Ferritin	465 ng/mL	10.0 – 150.0 ng/mL	Increased
HIV-1 RNA viral load	1650 copies/mL	0 copies/mL	Increased
CD4 T-lymphocyte count	286 cells/mm^3^	677-1401 cells/mm^3^	Decreased
CD4%	28%	38-55%	Decreased

**Figure 2:**
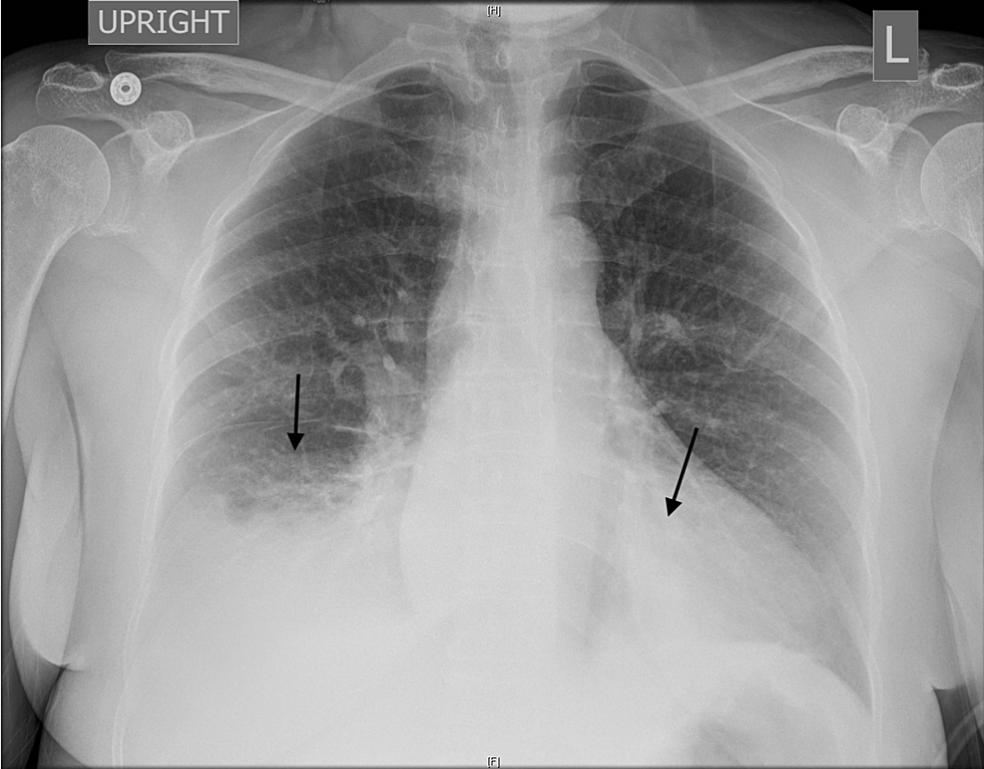
Chest radiography on the second presentation The left arrow depicts right basilar opacity, and the right arrow depicts retrocardiac opacity.

Admission was recommended for this immunocompromised patient with sepsis, but the patient left the ED to tend to a family emergency. Five days later, she returned to the ED for persistent fevers, worsening dyspnea, right-sided rib pain, cough productive of white sputum, and loose stools. Her temperature was 101.4°F, blood pressure was 155/85 mmHg, heart rate was 134 beats per minute, and oxygen saturation was 96% on ambient air. On physical exam, she was tachypneic, using accessory muscles, with expiratory wheezes heard on lung auscultation. A chest X-ray revealed a new, large, right basilar opacity and new, smaller, retrocardiac opacity, as depicted in Figure 2.

SARS-CoV-2 PCR was again negative. Other laboratory tests are depicted in Table 2.

**Table 2:** Patient’s laboratory values on the second presentation

Laboratory Test	Results	Normal Range	Interpretation
White blood cell count	12.7 k/uL	4.8 - 10.8 k/uL	Increased
Neutrophil count	14.9 k/uL	1.8 – 7.7 k/uL	Increased
Lymphocyte count	1.6 k/uL	1.8 – 4.8 k/uL	Normal
Monocyte count	0.5 k/uL	0.3 – 0.5 k/uL	Normal
Eosinophil count	0.0 k/uL	0.1 – 0.3 k/uL	Decreased
Basophil count	0.06 k/uL	0.0 – 0.05 k/uL	Increased
Serum sodium	122 mEq/L	135 - 145 mEq/L	Decreased
Blood urea nitrogen	7 mg/dL	5-20 mg/dL	Normal
Creatinine	0.8 mg/dL	<1.20 mg/dL	Normal

She was treated for community-acquired pneumonia (CAP) with ceftriaxone and azithromycin. She received systemic steroids and nebulized bronchodilators for an asthma exacerbation triggered by an acute infection. The leukocytosis was attributed to the receipt of systemic steroids on arrival at the ED. A urine *Legionella* antigen was sent on day 2 of her second admission (day 8 since the onset of symptoms) and returned positive the same day. No respiratory bacteriology cultures were sent at that time. She was ultimately diagnosed with Legionnaires’ disease caused by *Legionella pneumophila* serogroup 1. Therapy was switched to oral levofloxacin 750 mg daily to facilitate discharge. The New York City Department of Health and Mental Hygiene (DOHMH) was notified, and a public health investigation was initiated, which did not reveal an associated cluster of infections. A respiratory culture for* Legionella* was sent to assist with molecular typing. Further history revealed that the patient lived alone in an apartment complex in East Bronx, NY, which was originally built in the 1950s. She kept her air conditioning unit on throughout the day and had minimal recent contacts. She was not working at the time and received supplemental security income (SSI) benefits due to a disability. The patient clinically improved and was discharged home to complete a 14-day course of levofloxacin.

## Discussion

Legionnaires’ disease (LD) is categorized as an “atypical” community-acquired pneumonia. The species *Legionella pneumophila* is responsible for 80-90% of LD infections in humans, and it is primarily transmitted via aerosolization or aspiration [5]. Unlike SARS-CoV-2, *Legionella* is not transmitted between humans [5], and therefore isolation precautions are not required in hospitals or residential nursing facilities. The genus *Legionella* was first identified after an outbreak at the Philadelphia convention of the American Legion in 1976 [5]. It is an aerobic, gram-negative bacillus that naturally resides in aquatic bodies, such as lakes and streams, but can enter and proliferate in human-constructed water reservoirs. The species* Legionella pneumophila* can form microcolonies within biofilms, an important consideration for the decontamination of reservoirs in residences, hospitals, and older edifices [5].

Clinical manifestations of LD are nonspecific and difficult to distinguish from other etiologies of pneumonia, however, certain findings are more specific for LD and can serve as diagnostic clues, including diarrhea, high fever, hyponatremia, kidney injury, liver inflammation, failure to respond to beta-lactam antibiotics, and patient residence in a location where the water supply is known to be contaminated with *Legionella* [5]. Hyponatremia was present on the patient’s initial ED visit and was worse on subsequent presentation, which prompted the team to send *Legionella* testing, as this laboratory abnormality is less commonly associated with COVID-19. Almost all patients presenting with LD will have abnormal chest radiographs, but the findings are nonspecific. Table 3 offers a comparison between the key features of COVID-19, LD, and typical bacterial pneumonia caused by *Streptococcus pneumoniae* (pneumococcal pneumonia).

**Table 3:** Comparison between COVID-19, Legionnaires’ disease, and pneumococcal pneumonia SARS-CoV-2: severe acute respiratory syndrome coronavirus 2; FDA: Food and Drug Administration; NIH: National Institutes of Health; e.g.: exempli gratia; CAP: community acquired pneumonia; mRNA: messenger ribonucleic acid; U.S.: United States; CDC: Centers for Disease Control

	COVID-19	Legionnaires’ disease	Pneumococcal pneumonia
Common Presenting Symptoms	Variation in symptoms may depend on the variant of virus and phase of infection; initial symptoms may include cough, headaches, myalgias, diarrhea, loss of taste/smell, nasal congestion, sore throat, and sneezing, whereas progression to pneumonia may cause fever, cough, dyspnea	Fever, cough, dyspnea, diarrhea	Fever, cough, dyspnea
Primary Modes of Transmission	Transmission of the SARS-CoV-2 virus from human to human by respiratory droplets or aerosolized particles containing the SARS-CoV-2 virus	Aerosolization or aspiration from a contaminated water source; no confirmed human-to-human transmission	Colonization of the nasopharynx and subsequent aspiration of nasopharyngeal secretions
Common Lab Abnormalities	lymphopenia (variable total white blood cell count); elevated lactate dehydrogenase; elevated inflammatory markers (ferritin, C-reactive protein, erythrocyte sedimentation rate, interleukin-6)	Leukocytosis; hyponatremia; elevated blood urea nitrogen and serum creatinine; abnormal liver tests	Leukocytosis; bacteremia; anemia; mildly abnormal liver tests; hypoalbuminemia
Chest radiography	May be normal initially, however with progression to pneumonia common findings include bilateral, peripheral, lower zone consolidations and/or ground glass opacities; pleural effusion uncommon	Almost all patients will have abnormal chest x-ray with pulmonary infiltrates; pleural effusion may be present; findings generally nonspecific	Frequently abnormal, may range from a subtle change to dense consolidation; pleural effusion may be present
Treatment	Supportive care; multiple SARS-CoV-2 antiviral agents and/or immunomodulatory agents approved or authorized under emergency use by the FDA; most updated recommendations based on NIH COVID-19 Treatment Guidelines [6]	Macrolide or fluoroquinolone antibiotics	Empiric CAP therapy (e.g., intravenous ceftriaxone); if the diagnosis is confirmed then directed therapy can be tailored according to local antibiograms
Prognosis if untreated	Variable; the disease can be self-limited if mild-moderate; in severe disease, it may progress to respiratory failure and death	Poor; if untreated can lead to respiratory failure, septic shock, death	Poor; if untreated can lead to respiratory failure, septic shock, death
Preventive Measures	Physical distancing, face mask, hand washing, vaccination	Water management programs in residential and commercial facilities, hospitals, and nursing residences	Vaccination (see below)
Vaccine	Multiple vaccines approved or authorized in the U.S. by the FDA, including 2 mRNA, 1 viral vector, and 1 protein subunit vaccine; the most updated guidance can be found at the CDC website [7]	No vaccine available or in clinical trials	Pneumococcal polysaccharide vaccine and conjugate vaccine are widely available

LD is diagnosed via a urinary antigen test (UAT). Given the relatively low prevalence nationally, the Infectious Diseases Society of America (IDSA) recommends against routine UAT in most adults with CAP, unless indicated by epidemiological factors or in the case of severe pneumonia. In our case, the well-documented increased prevalence of LD during the summer months in the Bronx [8] was an indication of *Legionella* testing. When testing is indicated, *Legionella* UAT (up to 100% specific and 70-80% sensitive for *Legionella pneumophila *serotype 1) is recommended. *Legionella* culture of lower respiratory tract secretions or *Legionella *nucleic acid amplification testing can be performed as well, if available. Culture requires special media for recovery (buffered charcoal yeast extract agar) and is a useful epidemiologic tool to conduct molecular typing during outbreak investigations [7]. Targeted treatment requires antibiotics that will achieve high intracellular concentrations within alveolar macrophages, as *Legionella* is an intracellular pathogen. The treatment of choice is a macrolide (such as azithromycin) or a respiratory fluoroquinolone (such as levofloxacin). Observational studies suggest no significant differences between azithromycin and levofloxacin in terms of time to defervescence, time to clinical stability, or mortality [9]. While no optimal duration of therapy is established, extended courses of up to 14 days are recommended for patients who are immunocompromised or have a severe disease [1].

Legionellosis is a nationally notifiable disease to public health authorities. While most LD cases are sporadic, multiple recent outbreaks have been reported in urban centers throughout the United States with older residential and commercial infrastructure and contaminated water systems. In NYC, the DOHMH investigates every LD case by reviewing the medical record, interviewing patients and their close contacts, and obtaining clinical isolates for typing or genetic sequencing to establish an exposure source [8].

LD outbreaks disproportionately impact communities of color, living in poverty with decreased access to safe housing and affordable healthcare. Large-scale outbreaks in South Bronx, New York, and Flint, Michigan, exposed the vulnerability of poor communities to transmissible diseases [8]. The deteriorating infrastructure of water distribution systems, poorly maintained plumbing, and the use of low-flow water fixtures allow the proliferation of *Legionella* bacteria [4]. Likewise, the COVID-19 pandemic has uncovered similar vulnerabilities in people living in poverty or from historically marginalized racial and ethnic groups [3]. The contributing factors may be multifactorial, as people from these populations more commonly have chronic medical conditions, pre-disposing them to pulmonary infection, may live in population-dense areas or multigenerational households, rely more on public transportation, have less access to healthcare, or may hesitate to engage with the healthcare system [3]. While SARS-CoV-2 and *Legionella* are separate causative entities of pneumonia, they both underscore the disproportionate impact on the most vulnerable communities. Therefore, understanding a patient’s social determinants of health plays an important role in risk stratification for infectious diseases. Mitigating contributing factors would involve the concerted effort of clinicians and the local, state, and federal public health community.

We present the case of a woman from the Bronx, NY, who came to the ED in the summer of 2020, a few months after the peak of the first COVID-19 pandemic wave, with a febrile respiratory illness and was diagnosed with Legionnaires’ disease. She had risk factors for predisposition to both COVID-19 and LD, which included residence in the Bronx, tobacco use, and an immunocompromising condition (HIV). Her social history, which is an often-neglected aspect of a patient’s initial history, provided important diagnostic clues. She had not recently been exposed to other people and was confined to her apartment, which made COVID-19 less likely. Her air-conditioner was running continuously due to a recent heat wave, which in combination with her residence in an older building in the Bronx, was a clue for possible exposure to *Legionella*. Notably, her infection was sporadic and not associated with a cluster of other infections, which underlines the point that not all people exposed to *Legionella *will develop the disease. Her chest radiograph, which demonstrated right basilar and retrocardiac opacities, was not typical of pneumonia caused by COVID-19. Her initial diagnostic testing was notable for hyponatremia; although not specific for LD, it appropriately triggered the clinicians to send a *Legionella* UAT and arrive at the correct diagnosis.

## Conclusions

Our understanding continues to evolve regarding SARS-CoV-2 and the clinical manifestations of its numerous variants. It has emerged as an important cause of pneumonia and should be part of the differential diagnosis for any patient who presents with a febrile respiratory illness. The clinical syndrome alone cannot be used to distinguish between numerous causes of pneumonia as discussed in this case, such as SARS-CoV-2, *Legionella pneumophila*, and *Streptococcus pneumoniae*, given that the presenting symptoms and epidemiologic risk factors may overlap. As such, while the COVID-19 pandemic continues for the foreseeable future, other causes of febrile respiratory illness should be considered and investigated when appropriate.
